# Assessing infant motor development from afar: reflections on remote assessment of infant motor development

**DOI:** 10.1038/s41390-026-04767-1

**Published:** 2026-01-19

**Authors:** Maria Mc Namara, Kristian Budini, Esther Norfolk, Iona Novak

**Affiliations:** Specialty of Child & Adolescent Health, Cerebral Palsy Alliance Research Institute, Sydney Medical School, Faculty of Medicine & Health, The University of Sydney, Sydney, NSW Australia

## Abstract

**Abstract:**

The COVID-19 pandemic accelerated a global shift from face-to-face to digital healthcare, increasing demand for valid and reliable tools that can be administered remotely. The Alberta Infant Motor Scale (AIMS) is a widely used observational measure of infant gross motor development, yet evidence supporting its psychometric performance via telehealth remains limited. This study evaluated the validity of remote AIMS administration across three digital modalities compared with traditional in-person assessment. Findings demonstrated excellent agreement between remote and face-to-face scores, confirming that virtual administration provides clinically reliable information about infant motor development. Validating the AIMS for telehealth has substantial implications for equity, research, and clinical practice. Remote delivery reduces travel, cost, and logistical barriers, improving access to early developmental assessment for families in rural, remote, or low-socioeconomic contexts. Reliable remote assessment also enables broader research participation and supports hybrid clinical models that maintain continuity of care when in-person visits are not feasible. These results strengthen confidence in digital modes of assessment and highlight remote AIMS administration as a viable, scalable approach to early developmental surveillance. The findings offer particular benefit for infants who face the greatest barriers to timely evaluation and intervention.

**Impact:**

- Strengthens confidence in telehealth as a legitimate and scalable mode of early motor surveillance, especially for infants at risk of delay.
- Expands opportunities for equitable access by enabling high-quality assessment for families in rural, remote, or low-resource settings with barriers to in-person services.
- Supports inclusive and decentralised research models, enabling broader recruitment, reduced participant burden, and improved monitoring in clinical trials and developmental follow-up.

## Introduction

The COVID-19 pandemic changed clinical practice irrevocably on a global scale. Widespread lockdowns necessitated a rapid transition from face-to-face service provision to virtual platforms.^1^ This trend towards telehealth continues, resulting in a significant transformation in how healthcare is delivered. The increased flexibility, equity, and accessibility associated with increased use of digital healthcare modalities,^1–3^ are one of the unexpected silver linings to this otherwise devastating global health crisis. Digital platforms offer families more accessible and affordable treatment options, improving equity by reducing access barriers to healthcare services, particularly for underserved rural and remote communities.^2,3^ Traditionally, standardised motor assessments required in-person administration, allowing clinicians to adapt the environment, manage distractions, and tailor procedures to individual infant needs. However, widespread adaptation of such assessment tools for virtual administration has occurred.

Despite the surge in digital healthcare, there has been limited research conducted on the psychometric properties of assessment tools administered via digital platforms.^3,4^ Assessments, such as the Alberta Infant Motor Scale (AIMS), are valid and reliable tools for in-person administration,^3^ and have been adapted for use via digital platforms.^2,3^ However, to date, limited validation studies have been conducted.^3–5^

With the rapidly increasing uptake of digital healthcare, it is imperative to determine whether digital modes of service delivery are actually alleviating disparities or, conversely, compromising quality of care. Validation of a variety of modes of remote digital administration of assessment tools is critical to ensure that remote assessment is valid and reliable, and consistent with the ‘gold standard’ face-to-face delivery. Studies such as Santos et al., which evaluate three different modalities in a Brazilian setting, are a necessary and valuable contribution to the existing international literature.^3,6,7^

## The Alberta Infant Motor Scale

The AIMS is a norm-referenced observational assessment tool used to evaluate gross motor development trajectories in infants from birth to 18 months of age.^5,8,9^ The assessment lasts about 20–30 min, and it comprises 58 items across 4 subscales: prone, supine, sitting, and standing.^5,8,9^ Weight-bearing, posture and antigravity movements are assessed.^8^ The tool shows excellent psychometric qualities when used face-to-face in the Canadian context (Intraclass coefficient >0.82), where it was first developed.^3,5,8^ The AIMS has been strongly correlated with the motor domains of two well-established tests, the Bayley Scales of Infant Development and the Peabody Developmental Motor Scales.^8^

The psychometric properties of the AIMS when used outside of Canada are less well established.^5^ A recent systematic review summarising 49 studies (including 2 studies of remote video administration^6,7^) from 22 countries, investigated the use of the AIMS in international populations outside Canada.^5^ Overall, the authors reported acceptable reliability, discriminant validity, and concurrent validity across non-Canadian countries and criterion standard tests.^5^ However, the evidence ranged in quality from very low to high, with just five countries using culturally adapted versions of the AIMS with validated country-specific normative data. In particular, the AIMS predictive validity was found to be limited, showing variation across nations, criterion standard tests, and outcomes.^5^ For the most accurate infant developmental assessment, it is recommended that cross-culturally adapted versions of the AIMS are used with validated norms, where available.^3,5^ Further research is required in this regard for most locations.^3,5^ However, within the Brazilian context, existing evidence supports the use of Canadian-based norms,^5^ thereby further strengthening Santos et al.’s^4^ conclusions regarding the comparability of the AIMS’ psychometric properties across assessment modalities.

The AIMS is widely used internationally in both research and clinical settings to track motor development across diverse infant populations, including typically developing infants and those at risk due to medical history, e.g. infants born preterm, infants with neonatal encephalopathy, and infants admitted to the Neonatal Intensive Care Unit. The tool facilitates early detection of gross motor delay and timely provision of early intervention to optimise outcomes.^8–10^ The AIMS is shown to have the greatest sensitivity when administered to infants between 4 and 12 months of age,^9^ achieving 86% predictive accuracy for abnormal motor outcomes, supporting its use in cerebral palsy screening after 5 months of corrected age.^10^

## Significance

Validating the AIMS for telehealth and video-based administration carries substantial significance for equity, research, and clinical practice.^3–5^ As paediatric health systems increasingly adopt hybrid and remote models of care, ensuring that core developmental assessment tools maintain accuracy across modalities is essential. Santos et al.^4^ add to the existing literature^3,6,7^ to demonstrate that AIMS scores obtained via synchronous telehealth and asynchronous video recording show excellent agreement with traditional face-to-face assessments, confirming that remote formats provide clinically reliable information about infant motor development. Establishing this validity strengthens confidence in remote assessment as a legitimate and scalable component of early childhood developmental surveillance across cultural contexts.

From an equity perspective, validated telehealth assessments can meaningfully reduce long-standing barriers to accessing paediatric rehabilitation.^1–3^ Families living in rural and remote regions, those with limited transportation, and households facing financial or time constraints often struggle to attend in-person appointments. Remote AIMS administration reduces travel burden, minimises out-of-pocket costs, and supports participation for caregivers with work, childcare, or mobility limitations.^2,3^ By enabling high-quality assessment directly within the home environment, telehealth can widen access to early identification of developmental delays—an essential step in preventing widening gaps in child health outcomes among low-SES, geographically isolated, or otherwise underserved communities.^1–3^

The implications for research are equally important. Remote administration allows more diverse families to participate in studies, including those who may have been underrepresented due to distance from specialist centres.^3^ The ability to collect valid motor assessments remotely enables decentralised research designs, hybrid follow-up, and more frequent monitoring without increasing participant burden. This is particularly relevant for trials involving preterm infants or infants at higher risk of motor delay, for whom intensive observation schedules improve detection of developmental disabilities.^10^ Valid remote tools also enable large-scale, multi-site, and international studies, reducing logistical and financial barriers to recruitment.^3^

For clinical practice, integrating validated telehealth options expands service delivery models beyond traditional clinic-based care.^2,3^ Remote AIMS assessments allow clinicians to monitor progress between in-person visits, support early referral for intervention, and maintain continuity of care during disruptions such as illness, natural disasters, or pandemics.^3^ Robust remote monitoring options for infant motor development will further support early identification, timely referral, and continuity of care, particularly when frequent in-person visits are challenging or unaffordable. Together, these advantages position validated telehealth assessment as a critical component in improving equity, strengthening research inclusivity, generalisability, and recruitment, and modernising paediatric clinical practice.

## Conclusion

This study offers novel evidence that the AIMS can be administered remotely with excellent agreement to face-to-face assessment. Validating digital administration broadens the reach of early developmental surveillance and supports accurate monitoring when in-person visits are difficult.

Importantly, these findings have the greatest potential benefit for infants from socio-economically disadvantaged families and those living in rural or remote regions, for whom early access to assessment and intervention is often limited. By strengthening the evidence base for remote AIMS administration, this work contributes to more equitable, timely, and effective care for the infants who need it most.
